# Critical Time Intervention for People Leaving Shelters in the Netherlands: Assessing Fidelity and Exploring Facilitators and Barriers

**DOI:** 10.1007/s10488-015-0699-9

**Published:** 2015-11-16

**Authors:** Renée de Vet, Danielle A. M. Lako, Mariëlle D. Beijersbergen, Linda van den Dries, Sarah Conover, Albert M. van Hemert, Daniel B. Herman, Judith R. L. M. Wolf

**Affiliations:** 1 Impuls - Netherlands Center for Social Care Research, Department of Primary and Community Care, Radboud university medical center, P.O. Box 9101, 6500 HB Nijmegen, Netherlands; 2 Silberman School of Social Work at Hunter College, City University of New York, New York, NY USA; 3 Department of Psychiatry, Leiden University Medical Center, Leiden, Netherlands

**Keywords:** Critical time intervention, Model fidelity, Homelessness, Intimate partner violence

## Abstract

International dissemination of evidence-based interventions calls for rigorous evaluation. As part of an evaluation of critical time intervention (CTI) for homeless people and abused women leaving Dutch shelters, this study assessed fidelity in two service delivery systems and explored factors influencing model adherence. Data collection entailed chart review (*n* = 70) and two focus groups with CTI workers (*n* = 11). The intervention obtained an overall score of three out of five (*fairly implemented*) for compliance fidelity and chart quality combined. Fidelity did not differ significantly between service systems, supporting its suitability for a range of populations. The eight themes that emerged from the focus groups as affecting model adherence provide guidance for future implementation efforts.

**Electronic supplementary material:**

The online version of this article (doi:10.1007/s10488-015-0699-9) contains supplementary material, which is available to authorized users.

## Introduction

For people leaving homeless or women’s shelters, the transition to community living can be challenging. Because much has to be arranged during this stressful time, people are often in need of practical and emotional support. They can no longer utilize shelter services, which are generally terminated after shelter exit, and most of them have few supports that they can rely on in their new living environment (Herman et al. 2007). Relationships with family members and other potential social supports may need to be repaired first and ties to professional supports in the community may be weak or not yet established due to waiting lists. As a result, people leaving shelters experience a discontinuity of support. Post-shelter services are, therefore, vital in preventing negative outcomes such as recurrent homelessness and re-abuse (Caton et al. 1993; McQuistion et al. 2014; Tan et al. 1995; Tutty 1996).

Critical time intervention (CTI) is a time-limited, strengths-based case management model designed to prevent adverse outcomes in vulnerable people at the time of a critical transition in their lives, such as following discharge from institutional settings (Herman et al. 2007). CTI facilitates community integration and continuity of care by ensuring that a person has enduring ties to their community and support systems during these critical periods. It has been recognized by the Substance Abuse and Mental Health Services Administration (SAMHSA 2006), the Public Health Agency of Canada (2009), and the Coalition for Evidence-Based Policy (2013) as an evidence-based practice (EBP). In the United States, this intervention has been found to be effective in preventing recurrent homelessness and re-hospitalization as well as reducing psychiatric symptoms and substance use after the transition from shelters, hospitals, and other institutions to community living in people with severe mental illness (Herman et al. 2011; Kasprow and Rosenheck 2007; Susser et al. 1997). Furthermore, CTI is a cost-effective alternative to usual care for mentally ill men moving from a shelter to the community (Jones et al. 2003).

Few evidence-based interventions for vulnerable people leaving institutional settings have been tested rigorously outside the United States (de Vet et al. 2013; Jonker et al. 2015). Before EBPs are widely implemented internationally, it is necessary to test whether they are effective, because most of these practices have been developed to address place- and time-specific social issues (de Vet et al. 2013). In addition, different nations usually have distinct systems of care, which may influence the effectiveness of interventions (Toro 2007). Differences between systems of care might require adaptations of an intervention during implementation. These adaptations should be consistent with the model, so that its active ingredients are preserved. By evaluating whether CTI is effective outside the United States, we could possibly add to the evidence base supporting that this intervention’s mechanisms of effect are not dependent on a particular social context or health care system. We initiated two multi-center randomized controlled trials (RCTs) to test the effectiveness and model fidelity of CTI for homeless people and abused women in the Netherlands.

In modern effectiveness research, the development and use of fidelity criteria is considered obligatory to asses model adherence, that is, the degree to which a given intervention has been implemented in accordance with essential theoretical and procedural aspects of the model (Bond et al. 2000; Hogue et al. 2005). Earlier research shows that faithfully implemented EBPs produce better outcomes. For example, high fidelity to assertive community treatment (ACT) and strengths-based case management has been found to have a positive effect on client-level outcomes (Cuddeback et al. 2013; Fukui et al. 2012; McHugo et al. 1999).

So far, only one study has published CTI fidelity scores (Olivet 2013). This study was conducted by the Center for Social Innovation (C4) to assess differences in implementation and client outcomes between face-to-face and online CTI training. Fidelity was measured with the CTI fidelity scale, a quantitative tool developed by Conover and Herman (2007). The CTI fidelity scale consists of 20 items, which are rated on a five-point scale ranging from *not implemented* to *ideally implemented*. Item-level ratings can be combined to compute an overall fidelity score (Conover 2012). In the C4 study, overall fidelity scores were calculated nine months after training and were based on compliance fidelity, which is the degree to which providers implemented the key elements of the CTI model (eight items), and chart quality, which measures how well the intervention was documented (four items). The 15 North American homeless service agencies that participated in the C4 study obtained an average overall score of three on the five-point scale, which corresponds to *fairly implemented* according to the CTI Fidelity Scale Manual (Conover 2012). The present study was designed to provide insight in the implementation of CTI practice in three different ways. Firstly, we also conducted a fidelity assessment, which would allow us to examine whether a similar fidelity score would be achieved in the Netherlands as was obtained by the C4 study in North America. Secondly, we set out to compare the level of fidelity between two distinct service delivery systems—services for homeless people and services for abused women—which was possible because the two RCTs on CTI employed the same ongoing training and monitoring efforts during the same period in each service delivery system (Lako et al. 2013). Earlier studies of the effectiveness of CTI have already demonstrated that the CTI model can be successfully adapted for several types of populations (Herman and Mandiberg 2010). However, the hypothesis that CTI is suitable for a range of populations would be supported further if similar levels of model fidelity could be obtained in different service delivery contexts with the same implementation approach. Lastly, we aimed to provide insight into facilitators and barriers to CTI practice by conducting focus groups with the case managers trained in CTI (referred to as “CTI workers”). This will provide important information on which key aspects should be paid attention to when implementing CTI.

The present study will answer the following three research questions: What is the fidelity of CTI for homeless people and abused women making the transition from shelters to community living in the Netherlands? Is it possible to obtain similar fidelity ratings in two distinct service delivery systems (i.e., services for homeless people and services for abused women) with the same implementation approach? And which factors may have facilitated or impeded CTI workers to adhere to the CTI model in these service delivery systems?

## Method

### Procedure and Participants

This study is part of two RCTs examining the effectiveness of CTI for adult homeless people and abused women who are about to move to housing in the community and are willing to accept case management services during and after shelter exit. The two RCTs were initiated by the Academic Collaborative Center for Shelter and Recovery. The 18 shelter organizations that participated in these trials were members of this platform. In the Netherlands, services for homeless people are operated in a service delivery system that is separate from services provided to abused women; these two distinct service delivery systems will be referred to as *services for homeless people* and *services for abused women* in the remainder of this article.

Participant recruitment began in December 2010 and was completed in December 2012. In total, we recruited 183 clients from 18 homeless shelters, who had been rehoused within 14 months of entering the shelter, and 136 clients from 19 women’s shelters, who had been victim to any violence committed by an intimate partner (intimate partner violence) or committed to protect or restore the family honor (honor related violence) and stayed in the shelter for at least 6 weeks before being rehoused. The trials comply with the criteria for approval by an accredited Medical Research Ethics Committee (aMREC). Upon consultation, the aMREC region Arnhem-Nijmegen concluded that these studies were exempt from formal review (registration numbers 2010/038 and 2010/247). The methods of the two RCTs have been reported elsewhere in more detail (Lako et al. 2013).

Written informed consent to share client charts with the research team was obtained before participants were randomly allocated to CTI or care-as-usual. To assess the intervention’s fidelity to the CTI model, we randomly selected a sample of 70 charts, stratified by service delivery system, from participants allocated to the experimental condition. [Socio-demographic characteristics of these 70 participants are presented in the online supplement to this article.] In the two trials, 164 participants were allocated to CTI. In July 2013, we assessed which client charts were available for the fidelity assessment. Fifteen participants allocated to CTI had never been assigned a CTI worker (*n* = 15) and, as a result, did not have a CTI client chart that could be included in the assessment. Reasons for not assigning a CTI worker were that participants refused to receive services after randomization (*n* = 8), organizations were unable to provide CTI due to full case-loads or participants’ place of residence (*n* = 5), or participants were mistakenly assigned to another case manager (*n* = 2). For 17 participants, who had been allocated to CTI in the last 6 months of recruitment, the intervention had not yet ended and their CTI workers were therefore unable to supply these clients’ charts. Earlier research has shown that implementation of an EBP with a sufficient level of fidelity takes time (Fukui et al. 2012; Rapp et al. 2010b) and, therefore, CTI workers were expected to adhere more closely to the model at the end of the study than at the beginning. Because we aimed to draw a sample of charts representative for the study period as a whole, we decided to create temporal balance by excluding charts from participants who had been allocated to CTI in the first 6 months of recruitment (*n* = 33). To select a sample from the remaining charts available (*n* = 99), a computer-generated list of random numbers was used.

### Tailoring the Model

**Fig. 1:**
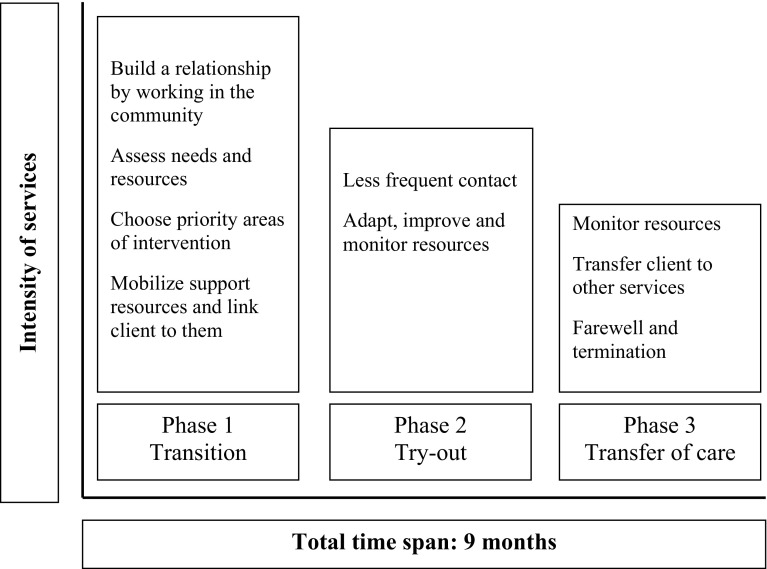
Intensity and focus of services during the three phases of the CTI model

CTI is divided into three phases, of 3 months each, with decreasing intensity of support over time (see Fig. 1). During the intervention the CTI worker provides practical and emotional support and helps to extend and strengthen the client’s social and professional network. Gradually, responsibility for the client’s care is transferred from the CTI worker to significant members from the client’s social and professional support system (Herman et al. 2007). Timing is crucial: An important principle of the model is that the CTI worker and the client have started building a working relationship before the actual transition begins (Herman and Mandiberg 2010).

When CTI was first introduced to the Netherlands, the model was adapted to enhance continuity of services for people with schizophrenia and a history of homelessness (Valencia et al. 2007). A pilot study tested the feasibility of implementing the adapted intervention. Adaptations were informed by data on housing instability among schizophrenia patients, interviews with clinicians and peer-specialists, and the investigators’ clinical and research experience with hard-to-engage populations (van Hemert n.d.). One of the adaptations was a more flexible time frame compared to the original model. A cardinal element in the CTI model is that the phase transition is automatically made at the three-month time point rather than driven by readiness criteria. In the adapted intervention, the time frame could be altered depending on the complexity of clients’ needs and problems, clients’ and case managers’ skills, and community factors, such as limited access to services due to waiting lists (Valencia et al. 2006). This adaption fits well with a growing interest in the Netherlands for the concept of providing personalized care (Evers et al. 2012). Decisions to transition to a subsequent phase were made by CTI workers and their supervisors during team meetings, which is an adaptation that was also incorporated in the implementation of CTI in the present study.

In addition to this model adaptation, implementation of CTI was also adapted to include elements from the strengths model (Rapp and Goscha 2011). Since most of the participating organizations had implemented a strengths-based approach to shelter services shortly before the start of the trial, principles from both the CTI and strengths model were integrated to ensure continuity in service approach during the transition from shelter to community. Because the strengths model stimulates clients’ capacity for autonomy and self-reliance by focusing on their strengths (Rapp and Goscha 2011), it is very compatible with CTI.

Besides modifications to improve the fit of the CTI model with the health care system and shelter services in the Netherlands, the intervention was also tailored to meet the special needs of women (and their children) who have experienced abuse. Although the idea for CTI was conceived in the mid-1980s when many people with psychiatric disorders were becoming homeless, this model also seems to suit the complex service needs of women who have experienced abuse. Earlier research has shown that when these women successfully obtain desired community resources and increase their social support, this will enhance their overall quality of life. This improvement in well-being appears to serve as a protective factor from subsequent abuse (Bybee and Sullivan 2002). We adapted the CTI model to employ practices familiar to the field, such as motivational interviewing (Millner and Rollnick 2002), and include a number of key components geared toward helping these women address and prevent problems that they and their children face. The original six CTI areas of intervention, which were selected because these had been identified as the most essential for treatment of people with a severe mental illness during a ‘critical time’ of transition, were adapted in consultation with managers and practitioners from shelter organizations in the Academic Collaborative Center for Shelter and Recovery. The final 10 areas of intervention were based on experiences of practitioners as well as literature on risk and protective factors for re-abuse. These factors were also incorporated in the Risk and Needs Assessment, a tool in the CTI client chart that helps to assess individual risks for recurrent homelessness and/or re-abuse and discontinuity of care.

### Training, Monitoring, and Support

Two or three case managers were drawn from existing staff of participating organizations to participate in the trials as CTI workers; they were generally part of service teams working in the community with vulnerable clients. Most of these case managers did not have any responsibilities within shelters. In order to qualify, staff members needed to have a bachelor’s degree in social work or a related field. In the fall of 2010, potential CTI workers were introduced to CTI by the research team and experienced trainers. The CTI workers completed three one-day training sessions to become familiar with CTI’s theoretical and procedural aspects and to acquire essential skills for CTI practice.

In addition to the initial training, CTI workers from all participating organizations attended centralized half-day training sessions—(bi)monthly during the first year and quarterly during the second year of study. With the aim of enhancing CTI practice, the research team and CTI trainer facilitated discussions in which workers from the participating organizations exchanged experiences, offered workshops on how to use CTI chart forms as tools for clients’ care, and invited CTI experts to present methods for enhancing CTI model fidelity.

Participating organizations were required to assign an internal coach, who was responsible for ensuring sufficient organizational support for the CTI workers and monitoring the model fidelity of the intervention. To this end, CTI workers had biweekly face-to-face supervision with their internal coach. Coaches received a one-day training session at the start of the trials and four half-day training sessions during the study period.

For the implementation of an EBP to be effective, leaders in an organization need to be committed to the change process (Brownson et al. 2012; McHugh and Barlow 2012). Several steps were taken to secure leadership buy-in. Firstly, the RCTs were initiated by the Academic Collaborative Center for Shelter and Recovery and designed in consultation with this platform’s steering committees and working groups, consisting of directors, managers, and practitioners from the member organizations. Secondly, each participating organization was visited at least twice by the research team before the start of the CTI training. During the first site visit, any possible challenges to the implementation of CTI were discussed with directors and managers. The second site visit was aimed at team leaders and practitioners to fill them with enthusiasm for the intervention. Lastly, presentations and workshops were conducted regularly at conferences and meetings to highlight the importance of the intervention, the trials’ objectives, and the study progress. The aim of organizing and attending these conferences and meetings was to ensure (continued) leadership buy-in of the participating shelters and policy makers in local government and other funding bodies.

### Fidelity Scale and Measures

Fidelity was measured with the CTI fidelity scale, a quantitative tool developed by two of the authors (Conover and Herman 2007). The CTI fidelity scale has been applied in a number of settings; however, this scale has not been formally validated so far (Herman and Mandiberg 2010). For the purpose of the two RCTs, the fidelity scale was adapted in consultation with the original authors and translated into Dutch. [The adapted version of the CTI fidelity scale and the rationale for each item in the original scale are available in the online supplement to this article.] Adaptations to the fidelity scale were for language as well as for elements from the strengths model. Items were not adapted to account for the planned change in the model with regard to flexibility in the time frame. Hence, the fidelity scale provided the opportunity to measure the deviation from the original model that resulted from this more flexible time frame.

**Fig. 2:**
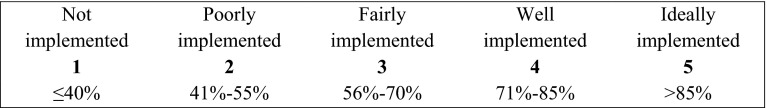
Conversion of percentages of positively rated criteria into five-point scale ratings

Each item of the CTI fidelity scale consists of one to five criteria, which can be rated positively or negatively. In order to obtain fidelity ratings at item-level, the number of positively rated criteria is divided by the total number of criteria to calculate percentages. These percentages are then converted into a five-point scale rating (see Fig. 2). Finally, all item-level ratings are added up, divided by the number of fidelity items, and rounded to the nearest integer to compute an overall fidelity score (Conover 2012).

The 20 items of the original CTI fidelity scale belong to one of three sections that each measure a different component of model fidelity: compliance fidelity, competence fidelity, and context fidelity (Conover 2012). The first section, compliance fidelity, is the degree to which workers practiced the key elements of the CTI model and is measured by eight items. Four of these indicate whether the intervention was delivered according to the intended CTI structure (i.e., as a nine-month intervention divided into three equal phases with a focus on up to three intervention areas): *Three Phases*, *Nine*-*Month Follow*-*Up*, *Time*-*Limited*, and *Focused*. The other four items are concerned with developing relationships with clients and their social and professional support systems: *Early Engagement*, *Early Linking*, *Outreach*, and *Monitoring*. The second section, competence fidelity, refers to the extent to which these key elements were delivered to clients with skill and attention to the craft (Fixsen et al. 2005) and is measured by nine items. Four of them rate how well the intervention was documented: *Intake Assessment*, *Phase Planning*, *Progress Notes*, and *Closing Note*. The other five items measure program quality: *Worker’s Role With Client*, *Worker’s Role With Linkages*, *Clinical Supervision*, *Fieldwork Coordination*, and *Organizational Support* (Olivet 2013). The third section, context fidelity, indicates whether the organizational requirements were met to allow the intervention’s practice to operate smoothly (Fixsen et al. 2005). Context fidelity is indicated by three items: *Caseload Size*, *Team Meetings*, and *Case Review*.

The organizations that participated in this study did not implement CTI throughout their organizations, but instead two to three CTI workers, who had other cases besides their CTI caseloads, operated independently within larger service teams for the benefit of the two RCTs. Due to this deviation in team structure and the small number of “active” CTI clients per worker at any time, conducting site visits as outlined in the CTI Fidelity Scale Manual (Conover 2012) was not appropriate. Therefore, the items that measure program quality (five items) and context fidelity (three items) could not be rated and were excluded from the assessment. The remaining 12 items of the CTI fidelity scale, which measure compliance fidelity and chart quality, were retained. The CTI Fidelity Scale Manual prescribes that these 12 items are rated by reviewing client charts.

### Fidelity Assessment

For the sample of 70 charts, we collected all the CTI chart forms that the CTI workers had completed. At a minimum, each CTI client chart had to contain an Intake Form, a Strengths Assessment, a Risk and Needs Assessment, an Activity Log, a Personal Recovery Plan for each phase, and a Closing Note. [The content and function of each CTI chart form are described in the online supplement to this article.] The Strengths Assessment and Personal Recovery Plan originate from the strengths model (Rapp and Goscha 2011); these chart forms were adapted to include elements that increase their compatibility with the CTI model and that are essential for their use during fidelity assessment (Wolf et al. 2012). CTI workers sent copies of the chart forms to the research team using postage-paid envelopes or e-mail. The research team tracked receipt of all forms in a password protected database. Digital copies of CTI chart forms were stored on a secure server and hard-copies of CTI chart forms were stored in locked cabinets.

Review of CTI chart forms and additional notes was conducted by two fidelity assessors, who were part of the research team and both had extensive knowledge of the CTI model. Agreement between assessors, derived from an independently rated subsample of 17 charts, was very high (Cohen’s κ = .80). The fidelity assessors used CTI fidelity worksheets (Conover 2012), which had been modified in line with the adaptation of the CTI fidelity scale, to record and rate the criteria of each item during chart review.

In addition to chart review, we conducted two focus groups with a convenience sample of CTI workers to assess which factors may have helped or hindered them to adhere to the basic components of the CTI model. The first focus group was conducted in February 2013 with CTI workers who supported abused women (*n* = 5) and the second group discussion was carried out in April 2013 with CTI workers who provided services to formerly homeless people (*n* = 6). Before the start of the focus groups, we obtained written informed consent from the participants. The questioning route was determined in advance and each focus group lasted approximately 110 min. During the interview process, the group moderator regularly restated or summarized information and then questioned the participants to determine accuracy. The group discussions were recorded and transcribed verbatim. Six weeks after the focus groups took place, meetings were organized to verify the results with CTI workers and internal coaches. Preliminary codes and themes, and carefully selected fragments from the focus group transcripts to illustrate these, were presented to the attendees, to which they could respond by correcting misinterpretations or adding more information.

### Analysis

For each item of the CTI fidelity scale, percentages of positively rated criteria were calculated at client-level using IBM SPSS Statistics for Windows, Version 20.0. Mean percentages for all client charts together and separately for services for homeless people and services for abused women were subsequently converted into fidelity ratings and an overall fidelity score for competence fidelity and chart quality.

Because fidelity ratings on separate items and the overall fidelity score could not be calculated at client-level, we tested for differences between services for homeless people and services for abused women before converting percentages into the five-point scale ratings. Mann–Whitney *U* tests were conducted to test for differences in percentages of positively rated criteria at item-level. An independent samples *t*-test was employed for the average percentage across all items. Because the group sizes are relatively small, and the analyses may lack statistical power as a result, we also calculated effect sizes.

Transcripts from the group discussions were explored using thematic analysis. The two lead authors (RV and DL) familiarized themselves with the data by listening to the recordings and rereading the transcripts. From one of the transcripts, they independently selected fragments considered to be relevant to the third research question. The supervising author (JW) reconsidered the relevance of extracted fragments and coded them inductively, developing an initial code frame. The lead authors used this frame to code the second transcript, using deductive and inductive analysis. To determine the validity of the information obtained and the code frame, a second data source was consulted, which consisted of questions and concerns about implementation from the CTI workers and internal coaches, and responses to these questions and concerns from the research team, collected during the study period. This document was continuously updated and disseminated during the centralized half-day training sessions. One of the lead authors (RV) combined the final codes into overarching themes, which were reviewed by two other authors (JW and MB). Existing themes were refined and finalized in consensus among the authors.

## Results

### Fidelity Ratings

Table 1 presents the percentages of positively rated criteria and fidelity ratings at item-level as well as the overall fidelity score for all client charts together (*n* = 70) and separately for services for homeless people (*n* = 35) and services for abused women (*n* = 35). Ratings of *Monitoring* (item 8) are based on a subsample of 63 client charts, because for seven clients—four clients from services for homeless people and three from services for abused women—the intervention had ended before phase 3 had begun. For all client charts together, the overall fidelity score for competence fidelity and chart quality is three out of five, which according to the CTI Fidelity Scale Manual indicates that fidelity to the CTI model is fair. On eight of the 12 items, CTI workers adhered fairly or well to the model; the other four items were not or poorly implemented.

**Table 1:** Percentages of positively rated criteria, fidelity ratings and overall fidelity score for all client charts together and each service delivery system separately

Fidelity scale items	All client charts together		Services for homeless people		Services for abused women	
	Percentage	Rating	Percentage	Rating	Percentage	Rating
Compliance fidelity						
Item 1: three phases	25	1	19	1	31	1
Item 2: nine-month follow-up	85	4	84	4	86	5
Item 3: time-limited	61	3	57	3	66	3
Item 4: focused	62	3	56	3	68	3
Item 5: early engagement	65	3	64	3	65	3
Item 6: early linking	65	3	67	3	63	3
Item 7: outreach	72	4	74	4	70	3
Item 8: monitoring	48	2	45	2	52	2
Chart quality						
Item 9: intake assessment	78	4	72	4	83	4
Item 10: phase planning	49	2	40	1	58	3
Item 11: progress notes	72	4	76	4	67	3
Item 12: closing note	36	1	40	1	32	1
Overall fidelity score	60	3	58	3	62	3

Ratings: 1 = not implemented, 2 = poorly implemented, 3 = fairly implemented, 4 = well implemented, 5 = ideally implemented

In relation to the intervention’s structure, CTI workers had generally divided the intervention into three phases, but failed most of the time to start and end each phase within a two-week margin of the intended three-phase structure. As a result, *Three Phases* (item 1) received a rating of 1, indicating this aspect of CTI had not been implemented. CTI workers scored well on *Nine*-*Month Follow*-*Up* (item 2), indicating that most of the time they managed to stay in touch with their clients for nine months and there were few major gaps where clients disappeared. They found it more difficult, however, to also end the intervention on time; *Time*-*Limited* (item 3) received a fair rating. A fair rating was also obtained on being *Focused* (item 4), which prescribes that the intervention should be limited to a maximum of three intervention areas.

With regard to relationship development, CTI workers should have met clients several times before shelter exit in order to gain an understanding of their clients’ histories; this *Early Engagement* (item 5) received a fair rating. *Early Linking* (item 6), which was also implemented fairly, prescribes that CTI workers maintain a high level of client contact during the first weeks after discharge and convene a joint meeting with family members and service providers to ensure continuity during this critical transition period. An element that was put into practice well is *Outreach* (item 7), which indicates that CTI workers regularly met in the community with clients and people in their support systems during phase 1. The poor rating on *Monitoring* (item 8) shows that, in phase 3, CTI workers had difficulty with adapting to their monitoring role; often, they met with or spoke to clients too frequently in that last phase.

With respect to chart quality, required sections of the Strengths Assessment and the Risk and Needs Assessment, which are both part of the *Intake Assessment* (item 9), and the *Progress Notes* (item 11) in the Activity Log had generally been completed; CTI workers scored well on these items. Unfortunately, *Phase Planning* (item 10) information on the Personal Recovery Plans was often incomplete. In addition, important elements were missing from the *Closing Note* (item 12) most of the time. These items were not or poorly implemented.

### Differences Between Service Delivery Systems

To compare the level of model fidelity between services for homeless people and services for abused women, we tested for differences in percentages of positively rated criteria at item-level and in the average percentage across all items. According to the independent samples *t*-test, the average percentage of positively rated criteria across all items did not differ between the two service delivery systems (*t*(68) = −1.42, *p* > .05). When percentages of criteria met at item-level were compared, we found a trend for three items (*p* < .10). CTI workers providing services to homeless people seem to be more careful to complete their *Progress Notes* (item 11; *U* = 461.50, *p* = .07), while CTI workers providing services to abused women seem to adhere better to the criteria regarding the *Intake Assessment* (item 9; *U* = 469.00, *p* = .06) and *Phase Planning* (item 10; *U* = 461.00, *p* = .06). For all three items, the effect size was small (*r* = −.22).

### CTI Workers’ Perceptions

The eight factors that emerged as prominent themes affecting model adherence are discharge and shelter services, working relationship, clients’ needs and attitudes, community support system, perceived effectiveness, model adaptation and trial design, organizational and team support, and tools and training. These themes are described below.

#### Discharge and Shelter Services

During the focus groups, CTI workers confirmed that continuity of care is crucial for a smooth transition from shelter to community living. Filling out an Intake Form together with a client and shelter case manager before discharge resulted in fewer loose ends once the client had moved. Most of the workers agreed that if they had been unable to engage clients before discharge, three months was too short for the first phase (*Three Phases*). Being assigned to clients who had already left the shelter made organizing a meeting with the client and shelter case manager (*Early Engagement*) more difficult, because often shelter case managers would be unavailable or clients too preoccupied, according to the CTI workers.

So after a month and a half I got [the meeting with the shelter case manager]. That’s how it works… Every time it’s like: The person that’s responsible is never there when you need them and that’s why it goes wrong all the time. And so I didn’t have any information from the client’s chart at all, so I just had to completely rely on the client at that point, and I really felt the lack of that meeting.

With regard to shelter services, working towards similar goals and with similar chart forms during shelter stay facilitated adherence to the CTI model. If clients had already completed, for instance, a Strengths Assessment in the shelter, then this version could be used by the CTI workers as a basis to expand from (*Intake Assessment*). CTI workers indicated that if clients had worked on strengthening their informal network with their shelter case manager, they seemed more willing to accept help in this area after discharge, as the following comment by a CTI worker reflects:

But one thing you can sort out [in the shelter], I think, has to do with their social network…. If the network doesn’t get mobilized while they’re in the shelter, then it’s very hard to mobilize it once they get their own place, because I’ve noticed clients are then like: I don’t need that any more.… So I think the time to seek help is in the shelter. If you engage [the network] at that point, then you can keep it involved later.

#### Working Relationship

In the CTI workers’ view, having a good working relationship with a client was also instrumental in model adherence. Workers indicated that it could take several weeks, or even months, to build a positive working relationship. Being able to engage clients early to start building a positive relationship was an important facilitating factor. CTI workers were very positive about having a meeting with clients together with their shelter case managers before shelter exit:

And the reason why that worked so effectively was… well, it gives the client a sense of safety, like: ‘Hey, my [shelter] case manager also thinks it’s a good thing that I’m going to start working with you.’ Quite primal, actually.

During the intervention, a trusting relationship between client and CTI worker appeared to be essential in helping to motivate clients. For example, several workers indicated that, even though some clients were reluctant at first, they had been successful in organizing a joint meeting with social supports (*Early Linking*) by following the client’s lead.

#### Clients’ Needs and Attitudes

Clients’ support needs, as well as their attitudes towards receiving support, also had an influence on model fidelity. Some clients, for instance, were quite hesitant to accept support from other professionals besides their CTI worker. Workers experienced that, even though other supports were available, certain clients would keep appealing to them, resulting in frequent contact during the last phase of the intervention. Workers also felt inclined to increase the intensity of the intervention if a client’s situation suddenly deteriorated, for example, due to an emotional or financial crisis. This could help to explain why the fidelity rating for *Monitoring* was poor. A crisis situation, however, could also motivate a client to become more accepting of help from others (*Outreach*), according to some of the CTI workers.

Halfway through the second phase, they discovered a spot on my client’s lungs. So then everything basically stood still for a while, but because of that, we did get to know his social network and could start drawing on that.

#### Community Support System

Model adherence also depended on workers’ success in developing community support. During the focus groups, CTI workers indicated that sufficient community support was necessary to allow them to decrease and eventually terminate contact with a client (*Time*-*Limited*) and that a client’s support system could help them gain more insight into a client’s situation and restore contact with a client when it had been disrupted due to frequent no-shows (*Nine*-*Month Follow*-*Up*). Several workers experienced difficulty linking clients to professionals due to austerity measures and this lack of access had hindered them in moving from the first to second phase (*Three Phases*):

[She] had an intellectual disability – or at least they [shelter staff] said ‘suspicions of’ – and as soon as she was home again [living in the community], you could tell. I basically ran into a brick wall trying to refer her. From pillar to post: Go there, try this and that. And at some point that frustrated her so much that she started rejecting everything.

Others experienced that, due to time constraints, professionals were often unwilling to attend joint meetings with a client’s support system, which according to the CTI model should be organized at the start and end of the intervention (*Early Linking* and *Closing Note*).

#### Perceived Effectiveness

Whether the workers perceived a certain component of the intervention as effective seemed to have had an influence on their willingness to adhere to the model. Several workers mentioned that CTI’s three-phase structure fitted well with their clients’ process of adaption to community living. For some of the workers, the decreasing intensity of CTI in the second and third phase meant they could spend more time with their clients during that first, crucial phase; they felt that they were able to match service intensity to their clients’ needs thanks to the implementation of the CTI model. During the focus groups, workers discussed how the time-limited nature of the CTI model had helped them change their mindset: They would make better use of supports in clients’ networks instead of providing support directly, especially in the second and third phase.

You’re already aware: During the first three months, I too will have to work very hard arranging and setting up practical things. But in the second phase you already start asking the client: ‘Okay, how would you do that, what things could you consider? Who can you turn to?’ And in the last phase you’ve resolved all that. You’re in a different position then.

The CTI workers expressed that, as a result, they had generally been comfortable with ending the intervention at nine months (*Time*-*Limited*).

CTI workers expressed far less motivation to adhere to model components that they did not regard as beneficial. For example, the model prescribes that CTI workers organize a transfer-of-care meeting at the end of the intervention (which should be documented in the *Closing Note*). The transfer-of-care meeting is a joint meeting during which significant members from the informal and formal support system, along with the client, reach a consensus about the components of such an ongoing support system. In the view of several workers, having such a transfer-of-care meeting was unnecessary, because each member’s role in the support system had already been discussed during a joint meeting in the first phase, and the system had been functioning well. Their perception of this element as redundant has most likely contributed to the poor fidelity rating for the *Closing Note*.

#### Model Adaptation and Trial Design

CTI workers mentioned that decisions about whether to move to a next phase with a client were made together with the internal coach and other CTI workers and were often based on a checklist of requirements for each phase—referred to as *anchor points*—(Wolf et al. 2012), which had been provided to them during the training sessions. The workers found this checklist a helpful tool in deciding whether they could move on to subsequent phases:

Something to fall back on [anchor points] is great, because that’s what you’re working to achieve…. But you’re also both aware – the client and the practitioner – that you’ve got that set amount of time to sort out those basic things…. So you just start to work. That’s great.

The decision to move a client to a subsequent phase was ultimately made by the CTI workers to enable them to provide personalized health care. This represents a considerable deviation from the CTI model, which most likely contributed to the poor degree of fidelity to *Three Phases*.

As mentioned before, CTI workers had other cases besides their CTI caseloads and operated independently within larger service teams. At the beginning of the recruitment period and at certain recruitment sites where few clients were eligible to participate, workers had few active CTI clients, which, according to the CTI workers, made it difficult for them to internalize the CTI model. Some of the CTI workers mentioned they were expected to have full standard caseloads at all times by their team supervisor. If a new participant had been assigned to CTI, they would often have to transfer clients receiving usual services to colleagues, or would sometimes be pressured to work overtime.

#### Organizational and Team Support

CTI workers indicated that generally they felt supported by their organizations, although organizational support was lacking in some organizations with respect to chart documentation. Several workers had to maintain a second client chart that met all of the organization’s standards, which may have resulted in less time spent on and lower quality of the CTI client chart. Furthermore, in one organization, standard procedures with regard to ending services after several no-shows were enforced for clients assigned to CTI, which directly contradicts with *Nine*-*Month Follow*-*Up*.

Having team meetings on a regular basis was crucial in adhering to the CTI model. According to the CTI workers, these meetings helped to reflect upon the delivery of the intervention and thereby reinforced activities that were consistent with CTI principles:

Reflection [during team meetings]. But also right in the middle of your work when someone suddenly reminds you: ‘Why haven’t you reached that point yet?’

Although the CTI model stipulates that team meetings should be organized every 2 weeks, several CTI workers mentioned that they met less often and did not feel properly supported by their internal coach. Reasons for having infrequent team meetings were having to travel large distances to meet, having too little time to meet due to full caseloads, and having little reason to meet due to the small number of active CTI clients.

#### Tools and Training

Having the right tools, and sufficient training to use them to a client’s advantage, facilitated adherence to the model as well. For example, CTI workers mentioned that the Personal Recovery Plan helped clients to set attainable short-term goals (*Phase Planning*), because clients had to indicate on a five-point scale how likely they were to achieve each goal in the next three months. Several workers mentioned that the ecogram, a tool to visually map support systems (Hartman 1978), proved to be helpful, especially with clients who relied heavily on their CTI workers. Drawing an ecogram together with the client made clear who else was available for support in their network, which, in turn, made it easier for the CTI worker to “pull back” when the intervention progressed (*Time*-*Limited*).

For example, I’d had a client make an ecogram…. Then I covered up somebody’s name with my thumb and said, ‘What happens if she’s not around?’ That was somebody who was to come hang the light fixtures. ‘Oh, well then I’ll get my uncle to come round.’ And then we did a few more, and at some point I put my thumb on my own name, and then she said something like, ‘Yeah… well perhaps I could phone my aunt sometime.’ And then it dawned on her: Hey, who could I call on then if he’s not available anymore?

## Discussion

The first and second aims of the study were to establish fidelity to the CTI model of an intervention for homeless people and abused women moving from shelters to community living in the Netherlands and to show whether it is possible to obtain similar CTI fidelity ratings in two distinct service delivery systems (i.e., services for homeless people and services for abused women) when the same implementation approach is employed during the same period. With an average of 60 % of positively rated criteria across all items, the intervention received an overall fidelity score for competence fidelity and chart quality of three out of five, which indicates CTI was fairly implemented according to the CTI Fidelity Scale Manual. This finding is similar to the overall fidelity rating in a previous multisite CTI study conducted with 15 service agencies in the United States and Canada (Olivet 2013). In the present study, the degree of fidelity on individual items ranged between not implemented (*Three Phases* and *Closing Note*) and well implemented (*Nine*-*Month Follow*-*Up*, *Outreach*, *Intake Assessment,* and *Progress Notes*). The two service delivery systems did not differ significantly on any of the items, although trends on three items related to chart quality were found. Effect sizes for these trends were small. This finding supports the hypothesis that CTI can be adapted for use with various populations, as suggested by Herman and Mandiberg (2010). Further research is needed to investigate whether this assertion holds when context fidelity and program quality, which are measured with the eight items from the CTI fidelity scale that were omitted in the present study, are taken into consideration. So far, however, the evidence seems to support that CTI’s context-sensitive timing is applicable to a range of service delivery systems that serve vulnerable populations. Perhaps, that is due to the fact that its program components were developed in collaboration with practitioners, which lead to a pragmatic intervention that may be somewhat atheoretical in nature (Jenson 2014).

The third study aim was to report CTI workers’ views on factors that may have facilitated or impeded adherence to the CTI model. From these factors, eight overarching themes emerged: discharge and shelter services, working relationship, clients’ needs and attitudes, community support system, perceived effectiveness, model adaptation and trial design, organizational and team support, and tools and training. CTI worker’s perceptions on factors that influence service delivery have been studied previously in a sample of 12 practitioners using CTI in a community agency or clinical trial setting in New York City (Chen 2012; Chen and Ogden 2012). Four of the themes that emerged in the present study—discharge and shelter services, working relationship, community support system, and organizational and team support—relate to the findings of this earlier study. Similarly to the CTI workers in the present study, practitioners interviewed by Chen (2012) stressed the importance of establishing contact with a client before the transition to a community residence. Not only have the benefits of early engagement been reported by practitioners, its effects on housing outcomes have also been empirically established (Herman et al. 2011). The present study corroborates the importance of fostering a trusting relationship to enhance client motivation and following clients’ leads as a practice strategy, as previously established by Chen and Ogden (2012). Furthermore, CTI workers at community agencies in New York City revealed making frequent use of their own agencies’ existing service programs (Chen 2012), which highlights the importance of easy access to community supports. In addition, they experienced that organizational policy occasionally conflicted with essential elements of the CTI approach, which was also the case in the present study.

Although the other four themes that emerged from the present study—clients’ needs and attitudes, perceived effectiveness, tools and training, and model adaptation and trial design—were not corroborated by earlier research on CTI practice, parallels can be drawn with findings from other studies of EBP implementation in mental health services. In a study conducted in child and adolescent mental health settings, clients’ concerns (for example, about the fit of an EBP with their own needs) and clients’ values were identified as factors affecting implementation (Aarons et al. 2009). In adult mental health services, clients have also expressed concerns that EBPs will result in limited choice in service options and less say in the specifics of their services (Scheyett et al. 2006). Integrating recovery principles with evidence-based interventions could be a good strategy to address concerns about the fit of EBPs with clients’ support needs and attitudes towards receiving support (Torrey et al. 2005). Concerning perceived effectiveness, Rapp et al. (2010a) identified practitioners’ resistance toward an EBP as a barrier to implementation at several community mental health centers; this initial resistance emanated from the practitioners’ assumptions about what works that contradicted with the EBP. Although the CTI workers participating in the present study were generally enthusiastic about the intervention, their assumptions did have a negative influence on their commitment to implement certain model elements. For instance, workers who deemed the transfer-of-care meeting to be unnecessary when the support system was functioning well, were unlikely to organize such a meeting at the end of the intervention. The importance of tools and training is addressed in another paper by Rapp et al. (2010b) which describes strategies for successful implementation of EBPs. The authors emphasize the importance of reinforcing the application of tools to achieve results, for example, by developing training units that focus specifically on the use of certain tools (such as the Strengths Assessment) in practice and by including tools in all aspects of systematic case review during team meetings. Regarding model adaption, CTI workers’ views on phase transitions, as well as the fidelity rating for *Three Phases*, pointed towards a deviation from the original model in the present study. This deviation, however, was in line with an a priori decision to adapt the model by focusing on readiness instead of making phase transitions automatically at each three-month time point. Fidelity scales can be a useful instrument in measuring model adaptation of EBPs, as illustrated by a study that focused on transferring clients from an ACT program to a less intensive adaptation of the ACT model (Salyers et al. 1998). Although programs which are more faithful to the original model have demonstrated better client outcomes, the need for adapting EBPs, which are generally developed in a particular socio-cultural and economic context, to local conditions has also been recognized (Bond et al. 2000).

In this article, we have distinguished eight factors that influence model fidelity. Whether other factors that have been identified previously as facilitators or barriers to EBP implementation also apply to CTI practice in Dutch shelter services, warrants further research.

### Strengths and Limitations

Together with an evaluation of a strengths-based intervention for homeless young adults (Krabbenborg et al. 2015), this study is the first to conduct a fidelity assessment of an evidence-based intervention in Dutch shelter services. Generally, few results from assessments of fidelity to the CTI model have been published (Herman 2014) and none of these previous studies have compared levels of model fidelity in two distinct service delivery systems. Moreover, this study contributes to a better understanding of model fidelity and implementation, because it combined quantitative and qualitative data to answer the research questions related to this topic rather than using either approach on its own (Robins et al. 2008). However, several limitations of the study need to recognized as well.

In the CTI Fidelity Scale Manual, cut-off points are provided to convert percentages of positively rated criteria into five-point fidelity ratings. In addition, norms are provided for how to interpret these ratings, ranging from *not implemented* (one out of five) to *ideally implemented* (five out of five). However, the CTI fidelity scale has not been formally validated so far and, as such, norms for good implementation have also not yet been established. Appropriate validation of the CTI fidelity scale is needed to determine whether the existing cut-off points and norms can be upheld.

Another limitation of the present study is that fidelity scores were calculated based on a subset of items from the original CTI fidelity scale. Because CTI was delivered to clients in a research context, participating organizations did not implement CTI throughout their organizations and the number of active CTI clients per worker was generally small. As a result, conducting site visits was not appropriate and eight of the 20 items of the original CTI fidelity scale, which measure program quality and context fidelity, had to be excluded from the fidelity assessment. If the omitted items would have been included, this could have altered the overall fidelity score as well as interpretation of the results. Inferences drawn based on the fidelity assessment are strictly limited to competence fidelity and chart quality and, based on these findings, no assumptions can be made about program quality or context fidelity of the intervention.

Nevertheless, valuable information about the context in which the intervention was delivered was obtained from CTI workers in focus groups. The use of focus groups, however, has certain limitations that should be highlighted, such as the possibility of social desirability and recall bias. Furthermore, data collected as the session progresses may represent opinions that are shaped by the group discussion (Carey 1995). The members of the group should, therefore, feel comfortable with each other. In the present study, focus group participants knew each other and the researchers well through the ongoing training sessions and were assured that the information they provided would be anonymously reported on. Therefore, we expect the data to accurately reflect the opinions of the focus group participants.

### Implications for Policy and Practice

The CTI fidelity scale and the assessment provide agencies and local policy makers with a framework for the development and quality assurance of EBPs that support vulnerable citizens during transitions in their lives. The identified facilitators and barriers to implementation might be used by policy makers and practitioners to improve fidelity to EBPs in shelter services and to provide the necessary conditions for successful implementation. Several recommendations for successful implementation of CTI can be made based on the study findings. First, staff should be committed to recovery and CTI principles, including the importance of fostering a good working relationship with clients. Important to model adherence is also their perception of the intervention’s components as effective. Assessing whether these core principles are part of the organization’s culture and the intervention’s components are integrated into work processes before implementation, and, if necessary, helping staff to internalize those principles through knowledge transfer (Rapp et al. 2010a), would be recommended. Sufficient access to a community support system is also important; CTI programs are unlikely to reach high fidelity in environments where access to informal as well as formal supports is very limited. In addition, CTI workers should be provided with sufficient organizational and team support as well as ongoing coaching. Coaching should foster mutual learning by reflecting together on the CTI model during regular case review and on the use of CTI chart forms as tools to improve clients’ care. Furthermore, workers should have full CTI caseloads to gain ample experience. Lastly, fidelity to the CTI model would improve if organizations integrate similar tools and principles in their residential shelter services and CTI workers are assigned at least several weeks before clients exit the shelter, which will enhance continuity of care during the transition from institutional to community living. In addition, training for shelter staff in how to enhance communication and collaboration pre-discharge could maximize the potential benefits from early engagement, as suggested by Chen (2012).

## Conclusions

This study shows that CTI was fairly implemented in the two multi-center RCTs testing the effectiveness of CTI for homeless people and abused women in the Netherlands. In these distinct service delivery systems—services for homeless people and services for abused women—the same implementation approach, employed during the same time period, resulted in very similar overall and item-level fidelity ratings. These findings are in line with the results from earlier studies that found CTI to be effective in different service delivery contexts: CTI seems to be an intervention suitable for a range of vulnerable groups who are going through a transition in their lives. Analyzing CTI workers’ perspectives on factors that may have influenced model fidelity has yielded important recommendations for successful implementation of CTI in other service delivery systems.

## Electronic supplementary material

Below is the link to the electronic supplementary material.

Supplementary material 1 (DOCX 37 kb)
