# Supplementary material for: Critical Time Intervention for People Leaving Shelters in the Netherlands: Assessing Fidelity and Exploring Facilitators and Barriers
Source: Adm Policy Ment Health. 2015 Nov 16;44(1):67–80. doi: 10.1007/s10488-015-0699-9 (PMC5225207; doi:10.1007/s10488-015-0699-9)
Supplement: Supplementary file 1 — Supplementary material 1 (DOCX 37 kb) [file 10488_2015_699_MOESM1_ESM.docx]

**Electronic supplementary material**

**Supplement 1** Socio-demographic characteristics of clients randomly selected for fidelity assessment

| *n* (%) | | all client charts together | services for  homeless people | services for  abused women |
| --- | --- | --- | --- | --- |
|  | | *n* = 70 | *n* = 35 | *n* = 35 |
| Mean (SD) age, years | | 37.9 (10.5) | 41.7 (10.8) | 34.1 (8.6) |
| Gender | |  |  |  |
|  | Male | 15 (21.4) | 15 (42.9) | 0 (0.0) |
|  | Female | 55 (78.6) | 20 (57.1) | 35 (100.0) |
| Migrant status | |  |  |  |
|  | Dutch | 34 (48.6) | 24 (68.6) | 10 (28.6) |
|  | First generation migrant | 29 (41.4) | 8 (22.9) | 21 (60.0) |
|  | Second generation migrant | 7 (10.0) | 3 (8.6) | 4 (11.4) |
| Marital status | |  |  |  |
|  | Divorced, widowed or never married | 49 (70.0) | 26 (74.3) | 23 (65.7) |
|  | Married or in civil union | 21 (30.0) | 9 (25.7) | 12 (34.3) |
| Children | |  |  |  |
|  | Has child(ren) | 57 (81.4) | 24 (68.6) | 33 (94.3) |
|  | Does not have any children | 13 (18.6) | 11 (31.4) | 2 (5.7) |
| Education level | |  |  |  |
|  | Low education level | 49 (70.0) | 24 (68.6) | 25 (71.4) |
|  | Intermediate education level | 13 (18.6) | 7 (20.0) | 6 (17.1) |
|  | High education level | 7 (10.0) | 4 (11.4) | 3 (8.6) |
|  | Unknown | 1 (1.4) | 0 (0.0) | 1 (2.9) |
| Psychological distress | |  |  |  |
|  | Elevated level of distress | 21 (30.0) | 13 (37.1) | 8 (22.9) |
|  | Average or low level of distress | 42 (60.0) | 21 (60.0) | 21 (60.0) |
|  | Unknown | 7 (10.0) | 1 (2.9) | 6 (17.1) |

**Supplement 2** The Dutch CTI fidelity scale, adapted from the original CTI fidelity scale developed by Conover & Herman (2007)

| Not  implemented | | Poorly  implemented | | | | | | | Fairly  implemented | Well  implemented | Ideally  implemented | | |
| --- | --- | --- | --- | --- | --- | --- | --- | --- | --- | --- | --- | --- | --- |
| **1** | | **2** | | | | | | | **3** | **4** | **5** | | |
| ≤40% | | 41%-55% | | | | | | | 56%-70% | 71%-85% | >85% | | |
|  | | | | | | | | | | | | % | R |
| ITEM 1 (Three Phases) | | | | Actual start & end dates (ASD, AED) are on Personal Recovery Plans, page 1.*  Projected start & end dates (PSD, PED) are calculated by fidelity assessor. | | | | | | | |  |  |
| Intervention occurs in 3 phases, and each phase lasts exactly 3 months (± 2 weeks). | | | | | | | | | | | |  |  |
| **1)** Start and end at least two Personal Recovery Plans on time. For each phase, assessor compares the ASD with the PSD, and compares the AED with the PED.  For Phase 1 (no projected start date):   - ends plan between 2 weeks before PED to 2 weeks after PED.   For Phases 2-3:   - starts each plan between 2 weeks before PSD to 2 weeks after PSD. - ends each plan between 2 weeks before PED to 2 weeks after PED. | | | | | | | | | | | |  |  |
| **2)** Start and end all three Personal Recovery Plans on time (to meet both criteria).* | | | | | | | | | | | |  |  |
| ** If only one Personal Recovery Plan is used throughout all three phases, find information about the ASD & AED in the Activity Log or in the CTI Monitor Record.* | | | | | | | | | | | |  |  |
| ITEM 2 (Nine-Month Follow-Up) | | | | | | | | Intervention actual end date (AED) is on Phase 3 Personal Recovery Plan or (if only one Plan for all 3 phases) on Activity Log. | | | |  |  |
| No dropouts. Staying in touch with clients is essential until nine-month time point (or 2 weeks earlier). Intervention is provided for most of this time. | | | | | | | | | | | |  |  |
| **1)** Be in touch with client at end of nine-month period (- 2 weeks) = 37 weeks.* | | | | | | | | | | | |  |  |
| ** Criterion #1 refers only to whether CTI workers are working with clients at the end. It does NOT refer*  *to the duration of their work, which can be from 37 weeks (criterion #2) to 41 weeks (item #3).*  *Calculation based on: (9 months x 4.3weeks/month = 38.7weeks) - 2 weeks = 36.7 or ~37 weeks.* | | | | | | | | | | | |  |  |
| **2)** Provide at least 7 months of intervention (-2 weeks) = 28 weeks.** | | | | | | | | | | | |  |  |
| *** Criterion #2 allows for breaks in the intervention over the nine-month period, but totaling more than 2*  *months. That is, when clients disappear or refuse to be seen in midst of intervention, this is not a problem as long as CTI workers continue to work with them at a later date.*  *Calculation based on: (7 months x 4.3weeks/month = 30.2 weeks) - 2 weeks = 28.2 or ~28 weeks.* | | | | | | | | | | | |  |  |
| ITEM 3 (Time-Limited) | | | | | Intervention actual end date (AED) is on Phase 3 Personal Recovery Plan or (if only one Plan for all 3 phases) on Activity Log. | | | | | | |  |  |
| The intervention never extends past nine-month time point (or 2 weeks later). | | | | | | | | | | | |  |  |
| **1)** Provide no more than 9 months of intervention (+2 weeks) = 41 weeks.* | | | | | | | | | | | |  |  |
| ** Calculation based on: (9 months x 4.3weeks/month = 38.7weeks) + 2 weeks = 40.7 or ~41 weeks.* | | | | | | | | | | | |  |  |
| ITEM 4 (Focused) | Intervention areas are on Personal Recovery Plan (page 1). | | | | | | | | | | |  |  |
| Work on linking clients to supports and monitoring those links during each phase is focused on only a few areas in order to ensure connections will be enduring & appropriate. | | | | | | | | | | | |  |  |
| **1)** For at least two of the Plans, identify no more than 3 intervention areas (not less than one) per Plan.* | | | | | | | | | | | |  |  |
| **2)** For all three Plans (to meet both criteria #1-2), identify no more than 3 areas (not less than one) per Plan.* | | | | | | | | | | | |  |  |
| ** If only one Personal Recovery Plan is used throughout all three phases, criteria #1-2 are met if that plan selects no more than three intervention areas on which to focus.* | | | | | | | | | | | |  |  |
| **3)** For at least two of the Plans, select intervention areas only from among the ten CTI areas listed on Plan.** | | | | | | | | | | | |  |  |
| **4)** For all three Plans (to meet both criteria #3-4), select intervention areas only from among the ten CTI areas listed on Plan.** | | | | | | | | | | | |  |  |
| *** If only one Personal Recovery Plan is used throughout all three phases, #3-4 are met if the intervention areas on that plan are all selected from among the ten standard intervention areas.* | | | | | | | | | | | |  |  |
| ITEM 5 (Early Engagement) | | | | | | | Information about meetings for criteria 1-2 is on Activity Log.  Information for criteria 3-5 is on Intake Form. | | | | |  |  |
| Meetings begin with clients as soon as possible to develop a close relationship with them. | | | | | | | | | | | |  |  |
| **1)** Hold first meeting with client within first month of pre-CTI phase  (e.g., if pre-CTI starts on May 5th, the first meeting with client is on June 5th at the latest). | | | | | | | | | | | |  |  |
| Meetings with clients at beginning are held frequently because assessment & engagement require intensive work. | | | | | | | | | | | |  |  |
| **2)** There is no more than 1 month* (=30/31 days) between each meeting with client during pre-CTI phase  and Phase 1. | | | | | | | | | | | |  |  |
| ** Due to variation in length of pre-CTI phase, frequency is not measured in # of times per phase for either pre-CTI or Phase 1.* | | | | | | | | | | | |  |  |
| Learning about client’s history needs to be a timely process in order to write first plan; give case presentation to team; and introduce client to new supports. | | | | | | | | | | | |  |  |
| Collaborate with client and shelter case worker on Intake Form to meet the following criteria: | | | | | | | | | | | |  |  |
| **3)** Fill out Intake Form some time during pre-CTI Phase until end of 6th week of Phase 1. | | | | | | | | | | | |  |  |
| **4)** Complete all required columns for almost all of the items (≥ 80%) on Intake Form. | | | | | | | | | | | |  |  |
| **5)** Obtain shelter caseworker’s signature (to ensure presence during completion of the form). | | | | | | | | | | | |  |  |
| ITEM 6 (Early Linking) | | | | Information about meetings is in Activity Log. | | | | | | | |  |  |
| Meetings with clients start early and are community-based to get to know them in their own environments. | | | | | | | | | | | |  |  |
| During the first two weeks of Phase 1 . . . | | | | | | | | | | | |  |  |
| **1)** Meet with:   - client at home   OR   - client at location where he/she receives services. | | | | | | | | | | | |  |  |
| Meetings with support network start early to meet existing supports, to introduce client to new supports, to introduce family members to providers or one provider to another. | | | | | | | | | | | |  |  |
| During Phase 1 . . . | | | | | | | | | | | |  |  |
| **2)** Meet with:   - client and at least one person from professional or social network   OR   - at least 2 people from the network (but not the client). | | | | | | | | | | | |  |  |
| ITEM 7 (Outreach) | | Information about meetings/calls is in Activity Log. | | | | | | | | | |  |  |
| Multiple contacts with clients & people in existing/potential support network occur in Phase 1. Most take place in the community - all assessments and majority of intervention activities are done where clients live, work, receive services, engage in recreation. | | | | | | | | | | | |  |  |
| During Phase 1 . . . | | | | | | | | | | | |  |  |
| **1)** Have at least 2 meetings with client in the community. | | | | | | | | | | | |  |  |
| **2)** Have a total of at least 3 meetings or calls* with client (any location). | | | | | | | | | | | |  |  |
| **3)** Have at least one meeting (any location) with:   - the person from whom client is renting his/her living accommodations   OR   - family member(s) or housemate(s) with whom client is living. | | | | | | | | | | | |  |  |
| **4)** Have at least one meeting (any location) with a professional from the network. | | | | | | | | | | | |  |  |
| **5)** Have total of at least 3 meetings (any location) or calls* with professionals or residential  caregiver/housemate. | | | | | | | | | | | |  |  |
| ** Do not count a call when the purpose is only to* ***schedule*** *a meeting.* | | | | | | | | | | | |  |  |
| ITEM 8 (Monitoring) | | | Information about calls and meetings is in Activity Log. | | | | | | | | |  |  |
| Monitoring means stepping back and having less frequent contact with client and supports. | | | | | | | | | | | |  |  |
| During Phase 3 . . . | | | | | | | | | | | |  |  |
| **1)** Call* or hold meetings with client no less than 21 days apart. | | | | | | | | | | | |  |  |
| **2)** Call* or hold meetings with a professional, residential caregiver or individual from social network no less than 21 days apart. | | | | | | | | | | | |  |  |
| ** Do not count a call when the purpose is only to* ***schedule*** *a meeting.* | | | | | | | | | | | |  |  |
| ITEM 9 (Intake Assessment) | | | | | | | Intake assessment information is on Risk & Needs Assessment form and Strengths Assessment form. | | | | |  |  |
| Risks, needs, strengths & resources should be assessed as soon as possible. | | | | | | | | | | | |  |  |
| **1)** Complete at least 80% of items on Risk & Needs Assessment form. | | | | | | | | | | | |  |  |
| **2)** Complete a Strengths Assessment form for at least 9 out of the 10 intervention areas. | | | | | | | | | | | |  |  |
| **3)** Date on Strengths Assessment form is before end of Phase 1.* | | | | | | | | | | | |  |  |
| ** For item 3, strengths & resources can be mapped for fewer areas than in item 2.* | | | | | | | | | | | |  |  |
| ITEM 10 (Phase Planning) | | | | | | Phase planning information is on Personal Recovery Plan. | | | | | |  |  |
| The CTI Plans help workers to provide an individualized intervention toward goal of abuse or housing loss prevention, which emphasizes strengthening support network. | | | | | | | | | | | |  |  |
| **1)** Record today’s date, phase start date and worker signature on Phase 1, 2 & 3 Personal Recovery Plans.* | | | | | | | | | | | |  |  |
| ** If only one Personal Recovery Plan is used throughout all three phases, this information must be recorded for Phase 1 and Plan must document all 3 phases.* | | | | | | | | | | | |  |  |
| **2)** Provide the rationale for selecting each area.** | | | | | | | | | | | |  |  |
| **3)** Describe at least one (short-term) goal for each treatment area.** | | | | | | | | | | | |  |  |
| *** If only one Personal Recovery Plan is used throughout all three phases, this information must be recorded for each treatment area in the Plan and Plan must document all 3 phases.* | | | | | | | | | | | |  |  |
| ITEM 11 (Progress Notes) | | | | | | Progress notes are in Activity Log. | | | | | |  |  |
| **1)** Complete at least 80% of required columns.  (contact with whom, when, etc.) | | | | | | | | | | | |  |  |
| **2)** Record all meetings in log that correspond to forms.  (Intake Form, Personal Recovery Plans, Risk & Needs Assessment, Strengths Assessment) | | | | | | | | | | | |  |  |
| **3)** Record (position within) institution for at least 80% of professionals. | | | | | | | | | | | |  |  |
| **4)** Record relationship to client for at least 80% of members of social network. | | | | | | | | | | | |  |  |
| ITEM 12 (Closing Note) | | | | | Information is on the Closing Note. | | | | | | |  |  |
| **1)** Holds and documents transfer-of-care meeting with client and at least one person from professional or social network during the last two months of the intervention. | | | | | | | | | | | |  |  |
| During exit meeting with client: | | | | | | | | | | | |  |  |
| **2)** Solicit feedback from client on CTI experience & discuss client’s progress since beginning of CTI. | | | | | | | | | | | |  |  |
| **3)** Discuss future (expected continuity of care & residential stability).  For abused women only: discuss expected safety. | | | | | | | | | | | |  |  |
|  | | | | | | | | | | | |  |  |

**Supplement 3** Rationale for the items in the CTI fidelity scale developed by Conover & Herman (2007)

|  | | **Item** | **Rationale** | **Dutch scale** | **Original scale** |
| --- | --- | --- | --- | --- | --- |
| **Compliance Fidelity** | | Three phases | The CTI intervention takes place in three phases, which each last no less than, and no more than, three months. | Item 1 | CMP4 |
|  |  | Nine-month follow-up | CTI workers make every effort to prevent people from dropping out of the intervention before the end of the nine months, and to locate people who have disappeared. | Item 2 | CMP8 |
|  |  | Time-limited | CTI is a cost-effective, time-limited intervention that serves to bridge the gap during the transition from institutional to community living, which means that it stops at nine months. | Item 3 | CMP7 |
|  |  | Focused | The intervention is limited to just a few areas which are crucial for enhancing continuity of care and for facilitating clients’ stability and community assimilation. | Item 4 | CMP5 |
|  |  | Early engagement | Before discharge, CTI workers gain understanding of clients’ histories and personalities, and start to build a rapport, in order to help clients adjust to expectations of community providers (and vice versa). | Item 5 | CMP1 |
|  |  | Early linking | CTI workers maintain high level of client contact during the first weeks after discharge, and convene a meeting with family members and providers, to ensure continuity during this critical transition period. | Item 6 | CMP2 |
|  |  | Outreach | Early in the intervention CTI workers establish linkages with anyone in the community who can help clients achieve stability in housing and an improved quality of life. | Item 7 | CMP3 |
|  |  | Monitoring | Because CTI workers make themselves obsolete during the intervention, they gradually reduce their role from directly delivering services to clients in phase 1 to low-intensity monitoring in phase 3. | Item 8 | CMP6 |
| **Competence Fidelity** | **Chart Quality** | Intake assessment | CTI workers carry out a comprehensive assessment of clients’ strengths as well as long-term housing, health care and psychosocial needs, which provides them with a familiarity with clients’ histories. | Item 9 | QUA1 |
|  |  | Phase planning | CTI workers formulate phase plans with a rationale for the decision to focus on a particular set of intervention areas (see item ‘Focused’) as well as general objectives for each intervention area. | Item 10 | QUA2 |
|  |  | Progress notes | For each relevant encounter or phone call, CTI workers add a progress note. Progress notes reflect the primary role of CTI workers, which is to connect clients to a community support system. | Item11 | QUA3 |
|  |  | Closing note | The closing note includes a summary of the intervention and the status of the network for assuring housing stability. It also records clients’ and community caregivers’ assessments. | Item 12 | QUA4 |

|  | | **Item** | **Rationale** | **Dutch scale** | **Original scale** |
| --- | --- | --- | --- | --- | --- |
| **Competence Fidelity** | **Program Quality** | Worker’s role with client | CTI workers develop a good rapport with clients by, among others, taking a ‘social solidarity’ approach to the relationship and being accessible to clients. | NA | QUA5 |
|  |  | Worker’s role with linkages | To help family members and/or providers become able to sustain coordinated and long-term support, CTI workers encourage communication, and negotiate if necessary, between clients and linkages. | NA | QUA6 |
|  |  | Clinical supervision | Supervisors frame their recommendations in the context of CTI principles in order to ensure that CTI workers adhere to the model. | NA | QUA7 |
|  |  | Fieldwork coordination | To enable effective case management and supervision, fieldwork coordinators regularly review charts for completeness, accuracy and quality, and remind the team where clients are in the intervention. | NA | QUA8 |
|  |  | Organizational support | The ‘parent’ organization in which CTI is implemented provides the resources and support that were promised at the start of a CTI program. | NA | QUA9 |
| **Context Fidelity** | | Caseload size | CTI workers’ caseloads are small (equivalent to a standard caseload of 18 or less), because they need to carry out an intervention that requires more time and effort than traditional case management. | NA | STR1 |
|  |  | Team meetings | By frequently meeting with all the CTI workers, supervisors can stay up-to-date with CTI workers’ activities and clients’ progress, provide expertise, and help CTI workers adhere to the CTI protocol. | NA | STR2 |
|  |  | Case review | Every CTI case is reviewed at least once every two weeks by someone trained in CTI supervision, in order to ensure that the CTI team stays on top of the intervention and status of every active client. | NA | STR3 |

*Note.* CMP = Components section, which measures compliance fidelity; QUA = Quality section, which measures competence fidelity; STR = Structure section, which measures context fidelity; NA = Not applicable to the Dutch CTI fidelity scale.

**Supplement 4** Contents of CTI client chart

CTI client charts consist of the following forms:

- The **Intake Form** contains a list of possible steps (in the areas such as finances, child care, employment, housing, etc.) that should be taken to prepare the client for the transition to community living and aids the CTI worker and client in deciding which actions are most urgent.
- A **Strengths Assessment** helps the worker and client to map the client’s unique strengths and resources.
- The **Risk and Needs Assessment** helps the worker to collect information about the client’s history and factors precipitating homelessness (or, in women’s shelters, factors precipitating victimization) as well as current professional and social support.
- In the **Activity Log**, the CTI worker registers with whom, where, when and how (e.g., face-to-face or by telephone) he or she had contact and what the purpose and duration of each contact was.
- A **Personal Recovery Plan** outlines the client’s goals, and means to reach these goals, in a maximum of three intervention areas for each phase. These intervention areas are chosen from ten life domains: security and protection against violence; finances and social security; relationship with (ex)-partner; employment and education; health and self care; living conditions and daily routine; children and parenting; social relationships; spare time and recreation; meaningfulness in life.
- The **Closing Note** contains a summary of the progress made by the client during the nine-month intervention, a description of the client’s current situation, a prognosis of the continuity of care and stability of housing, a report of the transfer-of-care meeting with client’s professional and/or social network, and a report of the exit-meeting with the client.
